# Usability and reproducibility of tear meniscus values generated via swept-source optical coherence tomography and the slit lamp with a graticule method

**DOI:** 10.1007/s10792-017-0517-3

**Published:** 2017-04-09

**Authors:** Hitoshi Imamura, Hitoshi Tabuchi, Shunsuke Nakakura, Daisuke Nagasato, Hiroaki Baba, Yoshiaki Kiuchi

**Affiliations:** 1 Department of Ophthalmology, Saneikai Tsukazaki Hospital, 68-1, Aboshi Waku, Himeji, 671-1227 Japan; 2 Department of Ophthalmology and Visual Sciences, Graduate School of Biomedical Sciences, Hiroshima University, Hiroshima, Japan

**Keywords:** Tear meniscus, Slit lamp, SS-OCT, Usability, Reproducibility

## Abstract

**Purpose:**

To investigate the usability and the reproducibility of the tear meniscus values via swept-source optical coherence tomography (SS-OCT) and the conventional slit lamp microscope method with a graticule.

**Methods:**

The right eye was examined in 90 healthy adult subjects who were grouped according to age (group 1: 20–39 years; group 2: 40–59 years; group 3: ≥60 years). The tear meniscus height (TMH) and tear meniscus area were measured using SS-OCT and TMH by the slit lamp microscope method. The reproducibility of each method was calculated using intraclass correlation coefficients (ICCs) in additionally enrolled 30 healthy young subjects. We also evaluated TMH at 3 mm from the corneal center in both temporal and nasal directions using SS-OCT.

**Results:**

The mean of the TMH values measured by SS-OCT was significantly higher than those measured by the slit lamp method (328 vs. 212 μm, *P* < 0.001, respectively). High reproducibility was observed for each method (ICC > 0.75 for both). No statistically significant differences were found in TMH among the age groups using both SS-OCT and slit lamp methods (*P* = 0.985, 0.380, respectively). TMH values at both sides of the corneal center were significantly smaller than those at the corneal center (*P* < 0.0001).

**Conclusions:**

TMH values obtained by the slit lamp method were lower than those obtained by SS-OCT. However, both methods yielded highly reproducible TMH measurements, suggesting that they are clinically useful. Tear meniscus values did not vary by age but by measurement points in our cohort.

## Introduction

Aqueous tear deficiency dry eye is generally characterized by lower tear meniscus values [1], whereas disorders of the lacrimal duct are generally characterized by higher tear meniscus values [2]. Tear meniscus measurement thus plays an important role in these diseases. Generally, most ophthalmologists subjectively check the tear meniscus height (TMH) on the lower eyelid using a slit lamp microscope as the first step to evaluate TMH. The attachment of a graticule to the slit lamp eyepiece increases the objectivity of TMH measurements [3–5].

In recent years, the clinical application of time domain anterior segment optical coherence tomography has dramatically increased the objectivity of tear meniscus measurements, including TMH and the tear meniscus area (TMA) [6–14]. The SS-1000 (Tomey Corp, Nagoya, Japan) swept-source OCT (SS-OCT) device, which was designed specifically for imaging the anterior segment, uses a wavelength of 1310 nm and allows for cross-sectional analysis as well as 3-dimensional analysis of the anterior segment of the eyes—thus enabling the measurement of TMH and TMA as well as tear meniscus volume via the use of an invisible long-wavelength light source that does not cause photophobia [10, 13]. SS-1000 is also capable of consecutive scanning of the tear fluid with a wide scanning range of 16 mm, including the center of the eyelid.

High reproducibility of the tear meniscus measurement by SS-OCT has been reported [10]. Additionally, TMH values or tear fluid volume by SS-OCT had the strong correlation between Schirmer test value [15]. However, in clinical practice, tear meniscus is evaluated mostly via the slit lamp and there is not a clear distinction between the role of OCT and slit lamp in tear meniscus evaluation. The first aim of our study was to first compare tear meniscus values obtained by SS-OCT to those obtained by the conventional slit lamp method with a graticule and further to investigate age-related changes in tear meniscus values by comparing the three groups created according to age of the subjects. The second aim was to evaluate the reproducibility of both methods. The third aim was to compare TMH at different measurements points (the center of the pupil and 3 mm from the center of the pupil in both temporal and nasal directions) using SS-OCT.

## Methods

### Subjects

This prospective, observational, cross-sectional study was performed in accordance with the Declaration of Helsinki and was approved by the Institutional Review Board of Saneikai Tsukazaki Hospital. A written informed consent was obtained from all healthy subjects. This study was registered with the Japan Clinical Trials Register; number: UMIN000005928. All of the measurements were taken in the right eye.

The exclusion criteria were subjects with ocular trauma, ocular inflammation, abnormal intraocular pressure, diabetic mellitus, a history of ocular surgery, any subjective symptoms or a diagnosis of dry eye, and/or any subjective symptoms or a diagnosis of epiphora. Exclusion criteria also included subjects who used contact lenses, eye drops within 24 h of the eye examination, and/or any topical or systemic medications that could affect tear secretion.

This study comprised two protocol sets. The comparative study between SS-OCT and slit lamp tear meniscus values and the study on age-related changes in tear meniscus values comprised 90 healthy adults in the age range of 20–86 years.

The reproducibility testing for SS-OCT and slit lamp comprised 30 healthy young subjects for each method.

### Tear meniscus measurements

All subjects underwent one anterior segment imaging using an SS-1000 device with 16 vertical raster scans at 1-mm intervals. One experienced examiner captured all of the images (H.B.). Each OCT image consisted of 512 A-scans with an acquisition time of 0.3 s. The axial resolution is approximately 8 µm, and the transverse resolution was approximately 30 µm. Subjects were instructed to look at a fixation light with no background light. The OCT image of right eye was taken 1 s after a natural blink [10, 13]. During the examination, the ambient room light, room temperature (25–28 °C), and humidity (40–50%) were maintained.

**Fig. 1:**
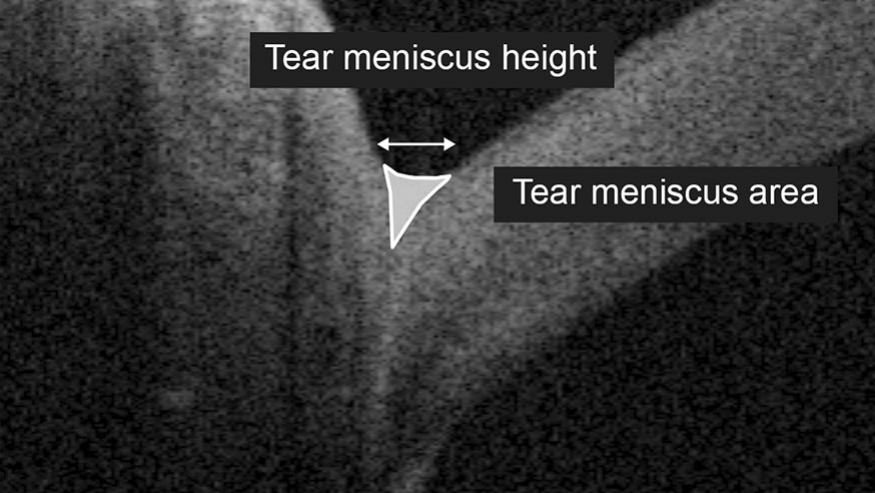
A tear meniscus image obtained by SS-OCT (male, 79 years of age). The *horizontal arrow* indicates the tear meniscus height (0.288 mm). The area within the manually *plotted lines* indicates the tear meniscus area (0.029 mm^2^)

Of 16 vertical raster scans, captured OCT images taken at the center of the pupil and 3 mm from the center of the pupil both in the nasal and temporal directions were magnified by 300%, and the TMH was measured using software calipers on vertical scans that were centered on the cornea. Then, for images taken at the center of the pupil, outlines of the tear meniscus were plotted using seven points with the software calipers (Fig. 1). The integrated software program calculated the area within the plotted outlines. To account for refraction at the air meniscus interface, the calculated value was then divided by the refractive index of a balanced salt solution (1.343) to obtain the TMA [10].

**Fig. 2:**
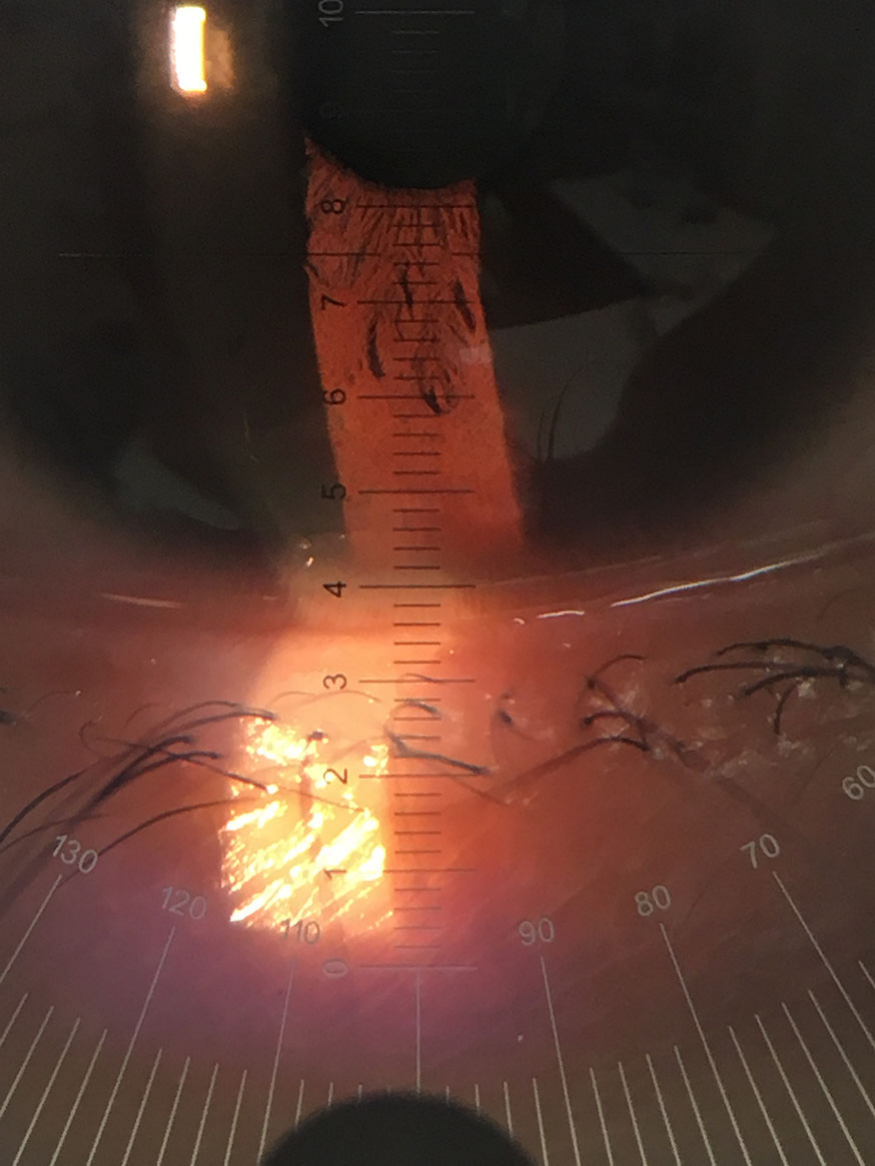
An anterior segment image obtained by the slit lamp method with a graticule (female, 30 years of age). One scale increment was equivalent to approximately 200 µm. In this case, the tear meniscus height was estimated to be 400 µm

After the OCT scan, the subjects underwent one TMH measurement using a slit lamp microscope. A graticule (Carl Zeiss #319770-9032-00, Oberkochen, Germany) was attached to the right eyepiece of the slit lamp microscope (Carl Zeiss SL130, Oberkochen, Germany). At 8× magnification, when observing the plane perpendicular to the observational axis direction, one scale increment was equivalent to 200 µm. All measurements were taken at 8× magnification at the center of the cornea without fluorescein staining. We read in 20 µm, which was 1/10 of the increment, for TMH (Fig. 2). We selected 8× magnification because the measurements of TMH at higher magnifications required more time and were considered inappropriate for clinical practice. Subjects were instructed to look straight with minimum observational illumination and were allowed to blink. The TMH of the right eye was measured by H.I. 1 s after a natural blink [10, 13]. The room light, room temperature, and humidity were similarly maintained as the OCT examinations.

### Comparison between SS-OCT and slit lamp tear meniscus values and the study on age-related changes in tear meniscus values

Ninety healthy subjects (age range, 20–86 years; mean age, 50.8 years; 43 males and 47 females) were enrolled in this study.

One set of TMH and TMA values measured by SS-OCT and TMH measured by slit lamp method was used for the subsequent analysis.

To investigate age-related changes in tear meniscus values, subjects were classified into three groups, with 30 adults per group, according to age: Group 1 was the young adult group (range, 20–39 years; mean, 29.6 years; 18 males and 12 females), group 2 was the middle-age group (range, 40–59 years; mean, 48.8 years; 12 males and 18 females), and group 3 was the elderly group (range, ≥60 years; mean, 74.1 years; 13 males and 17 females).

### Measurement reproducibility of the SS-OCT and slit lamp method

A group of 30 young healthy subjects (male, *n* = 11; female, *n* = 19) were additionally recruited to investigate the reproducibility of the tear meniscus measurements. Each subject underwent anterior segment OCT imaging three times. The captured OCT images were analyzed by two ophthalmologists (H.I. and D.N.) to obtain both TMH and TMA values.

Similarly, another group of 30 young healthy subjects (10 males and 20 females) were recruited, and the ophthalmologists (H.I. and D.N.) performed TMH measurements thrice using the slit lamp method. All measurements were taken in the right eye in one day. Based on the values obtained from these measurements, the inter-rater and intra-rater reproducibility of TMH and TMA measurement by SS-OCT and TMH measurement by the slit lamp method were investigated.

### Statistical analysis

Statistical analyses were performed using JMP software (version 10.0; SAS Institute Inc., Cary, NC). A one-way analysis of variance was performed to compare the three age groups.

Two types of intraclass correlation coefficients (ICCs) were used to assess the reproducibility of the measurements: ICC (2, 1), i.e., inter-rater reliability using a two-way random-effects model, and ICC (1, 1), i.e., intra-rater reliability using a one-way random-effects model. An ICC value of 0 indicates the level of agreement produced by chance alone, while a value of 1 indicated a perfect, positive agreement. We classified agreement, based on the ICC values, as poor (<0.4), good (0.4–0.7), and very good (>0.7), according to the principles of McGraw and Wong [16].

In addition, the coefficients of variance of TMH by the slit lamp method as well as TMH and TMA by SS-OCT were calculated. The correlation coefficients between TMH measured by the slit lamp method and SS-OCT as well as between TMH measured by the slit lamp method and TMA measured by SS-OCT were investigated. Multiple comparison tests (Bonferroni test) were used for comparisons among TMH values at different measurement points by SS-OCT. *P* values of <0.05 were considered statistically significant.

## Results

### Comparison between SS-OCT and slit lamp tear meniscus values and the study on age-related changes in tear meniscus values

The TMH value at the corneal center obtained by SS-OCT was significantly higher than that obtained by the slit lamp method [mean (SD); 328 (91) µm vs. 212 (50) µm, *P* < 0.001, paired *t* test] (Table 1).

**Table 1:** Inter-rater ICC (2, 1) and intra-rater ICC (1, 1) reliability in SS-OCT and slit lamp method

	ICC (2, 1)	ICC (1, 1)	
		Rater 1	Rater 2
SS-OCT			
TMH	0.932 (0.865, 0.966)	0.981 (0.965, 0.990)	0.932 (0.881, 0.964)
TMA	0.982 (0.956, 0.992)	0.990 (0.982, 0.995)	0.971 (0.948, 0.985)
Slit lamp			
TMH	0.761 (0.558, 0.878)	0.974 (0.954, 0.987)	0.853 (0.752, 0.921)

The values in parentheses indicate 95% confidence intervals

The mean (SD) of the TMH values measured by SS-OCT for each of the three age groups was: group 1, 327 (75) µm; group 2, 330 (95) µm; group 3, 326 (106) µm. The mean (SD) of the TMH values measured by the slit lamp was: group 1, 206 (41) µm; group 2, 209 (45) µm; group 3, 223 (61) µm. The range and median values of TMH in 3 groups measured by both methods are shown in Table 2.

**Table 2:** Range and median values of TMH in 3 groups (µm)

Age groups	Median	Maximum	Minimum
Group 1			
Slit lamp	200	300	120
SS-OCT	320	481	187
Group 2			
Slit lamp	200	300	120
SS-OCT	322	601	202
Group 3			
Slit lamp	200	340	100
SS-OCT	339	514	173

**Fig. 3:**
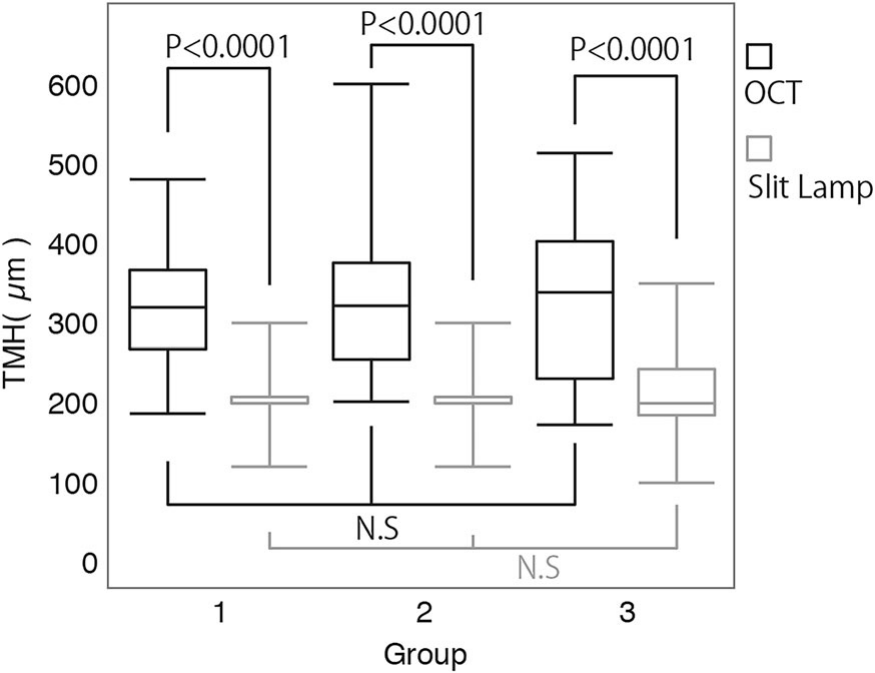
*Box plots* showing the distribution of tear meniscus height (TMH) measured via SS-OCT and slit lamp methods. No significant differences were found among the three subject groups using either method The TMH values measured by the SS-OCT were significantly higher than those measured by slit lamp method in all age groups (*P* < 0.0001)

There were no significant differences among the three groups regarding the analysis of variance for TMH measurements that were performed using SS-OCT (*P* = 0.985) or by the slit lamp method (*P* = 0.380). TMH values measured by SS-OCT were significantly higher than those measured by the slit lamp method in all age groups (*P* < 0.0001) (Fig. 3).

The mean (SD) of the TMA values measured by SS-OCT for each group was: group 1, 265 × 10^2^ (1380) µm^2^; group 2, 254 × 10^2^ (1110) µm^2^; group 3, 299 × 10^2^ (1700) µm^2^. The range and median values of TMA in 3 groups measured by SS-OCT are shown in Table 3. There were no significant differences among the groups as per the analysis of variance (*P* = 0.444). Table 3 shows the range and median values of TMA in 3 groups.

**Table 3:** Distribution of TMA Values in 3 groups (×10^2^ µm^2^)

Age groups	Median	Maximum	Minimum
Group 1	238	678	104
Group 2	246	551	89
Group 3	279	707	97

The correlation coefficient (*r*) between TMH obtained by the slit lamp method and TMH obtained by SS-OCT was 0.426 (95% confidence interval 0240–0.582, *P* < 0.0001), and that between TMH obtained by the slit lamp method and TMA obtained by SS-OCT was 0.457 (95% confidence interval 0276–0.606, *P* < 0.0001).

### Measurements reproducibility of SS-OCT and the slit lamp method

Table 1 shows the inter-rater and intra-rater reproducibility [ICC (2, 1) and ICC (1, 1), respectively] results. The inter- and intra-rater reliabilities of TMH measured by SS-OCT were slightly higher than those of TMH measure by the slit lamp method; however, both methods provided very good reproducibility (ICC > 0.7).

The coefficients of variance (CV) of TMH and TMA by SS-OCT were 0.28 and 0.52, respectively, and CV of TMH by the slit lamp was 0.23.

### Evaluation of TMH at different measurements points by SS-OCT

We were able to obtain clear SS-OCT images of TMH at 3 mm from the center of the pupil in both nasal and temporal directions for 70 subjects. In the other 20 cases, the images were unclear. Multiple comparison tests (Bonferroni test) demonstrated a statistically significant difference in the mean TMH between the center of the pupil [328 (91) µm] and the nasal side [290 (96) µm], between the center of the pupil and the temporal side [253 (71) µm], and between the nasal side and the temporal side (all, *P* < 0.05). TMH values at both sides of the corneal center were significantly smaller than those at the corneal center (*P* < 0.05).

## Discussion

In this study, the TMH values obtained by SS-OCT were significantly higher than those obtained by the slit lamp method. This is probably because SS-OCT imaging enables the observation of the tear film, which is produced by the surface tension effect and which cannot be detected by the slit lamp method. The slit lamp method may cause photophobia, which evokes the reflex lacrimation, but the effect of this reflex lacrimation is thought to be smaller for the difference. We also demonstrated that tear meniscus measurements using SS-OCT and the conventional slit lamp method have very good reproducibility (all, ICC > 0.7). In addition, moderate correlations were found between the TMH value obtained by the slit lamp method and the TMH and TMA values obtained by SS-OCT (*r* = 0.426, 0.457, respectively). Moreover, CV of TMH and TMA calculated from our data indicated that SS-OCT produced less variability in TMH (0.28) than in TMA (0.52). The slit lamp method showed less variability than the SS-OCT method. Therefore, TMH measurement using a slit lamp with a graticule may still be one of the most clinically useful methods to evaluate tear meniscus. In many previous studies, the slit lamp magnification of 32× was used for TMH measurement. The reason why 8× magnification was used in our study is because it is more clinically suitable. At 32× magnification, the patient’s movements have a larger influence on the measurements, and the measurements of TMH require more time. To ensure the appropriateness of the magnification setting, we measured TMH in 20 eyes of 10 healthy subjects at 8× and 32× magnifications, which revealed no significant difference [*t*(33) = −0.65, *P* = 0.52] (paired *t* test).

Previous studies conducted using the slit lamp method reported the TMH to be 120 [3], 190 [4], and 250 µm [5]. The procedures used in previous reports vary: capturing immediately after the blink [4] and unspecified capturing timing [3, 5], which may be responsible for a wide variation in values. In the present study, TMH was measured using a slit lamp microscope 1 s after the blink as the same way as using OCT in accordance with the preceding study [10, 13]. The mean TMH value obtained at the corneal center was 212 µm, which was nearly equivalent to the values presented in previous studies [4, 5]. Weak reproducibility of the TMH measurements by the slit lamp method was previously reported by Santodomingo-Rubido et al. [3] when comparing measurements ascertained at 6-month intervals; however, there have been no reports investigating ICC using the slit lamp method.

The TMH of healthy subjects measured by SS-OCT have been reported to be 256 µm [10], which is smaller than our result of 328 µm. Previous studies with time domain OCT have reported the TMH measurements in healthy subjects to be approximately 200 [9], 250 [7, 10], and 400 µm [8]. Although good reproducibility of TMH measured by time domain OCT was previously reported [17], the reason for such has yet to be identified, with values greatly varying from study to study.

Cui et al. [9] reported a significant decrease in the tear meniscus measurements obtained by time domain OCT due to aging, which was attributed to age-related reduction of lacrimal gland function (which results in a decreased tear secretion volume). However, the inclusion criteria for the study included a Schirmer I test value of more than 5 mm. Thus, there is the possibility that subjects with an age-related decrease in tear secretion were excluded from the study and that the age-related changes reported by Cui et al. might not accurately reflect the situation in normal subjects. Conversely, Patel and Wallace [4] measured TMH of healthy subjects without any tear-related disorders or symptoms using the slit lamp microscope method with a graticule eyepiece and reported a significant increase in TMH due to aging.

In contrast, we did not observe any significant differences in the TMH values among the three age groups of our study. We excluded subjects with either a history or any symptoms of dry eye or epiphora. We must consider the possibility that age-related physiological changes in lacrimal gland function may lead to a decrease in the tear meniscus values. Similarly, we should consider that age-related changes in lacrimal drainage function may lead to an increase in tear meniscus values. Changes in lacrimal drainage function involve many factors, including the decreased patency of the lacrimal duct [18], a decrease in the absorbing mechanism of the lacrimal duct, lacrimal drainage system failure caused by dysfunction of the orbicularis muscles, including Horner’s muscles [19]. Considering all of the possible age-related dysfunctions that could occur within each factor, it is possible that age-related lacrimal gland dysfunction and lacrimal drainage dysfunction may coexist to varying degrees. This could explain why there is a difference in age-related changes of tear meniscus values among the previously mentioned studies [4, 9].

Our study data demonstrated that TMH values measured at the nasal and temporal side of the center of the pupil were significantly smaller than those measured at the center of the pupil (290 vs. 328 and 253 vs. 328 µm; all, *P* < 0.05). The curve of the lower lid margin varies between individuals. We found that the TMH values in OCT images were higher at the lower point of the lower lid curve in about 70% of subjects. Some of the causes are likely to be fluid accumulation due to the influence of gravity or the role of the underlying tissue at the back site of the meniscus [20–23]; however, other factors, including the influence of tear flow in meniscus, should be further investigated. Regardless, one needs to be aware of the differences in TMH values depending on the measuring point on the lower lid curve.

Regarding limitations of our work, the exclusion criteria for healthy subjects included any symptoms that were suggestive of dry eye or a diagnosis of dry eye or epiphora. Thus, we cannot necessarily apply our results of this study to patients with dry eye or epiphora. These limitations could be addressed in future studies. In summary, the findings of the present study suggested that the slit lamp method, if used with a graticule, can yield comparable measurements to SS-OCT. We observed that TMH values obtained by the slit lamp method were lower than those obtained by SS-OCT. Additionally, tear meniscus values did not vary by age but by measurement points in our cohort. In large-scale clinical facilities, the OCT method may be appropriate for medical staff other than the attending doctor to measure TMH as it can be measured with less bias. Notably, the slit lamp method is also useful in general-scale clinics.
